# Parsonage-Turner Syndrome: An Unusual Cause of Postoperative Complications

**DOI:** 10.7759/cureus.93931

**Published:** 2025-10-06

**Authors:** Gretel Carmenate, Rhiya Mittal, Ralf E Gebhard, Keith A Candiotti

**Affiliations:** 1 Anesthesiology, University of Miami Miller School of Medicine, Miami, USA

**Keywords:** brachial plexus neuritis, idiopathic amyotrophic neuralgia, neuralgic amyotrophy, parsonage-turner syndrome, plexopathy

## Abstract

Parsonage-Turner syndrome (PTS), an uncommon and debilitating disorder, is a condition that can cause abrupt, severe unilateral shoulder pain followed by upper extremity sensory disturbances and weakness. On average, patients may experience significant upper limb weakness two to four weeks after pain onset, with weakness potentially lasting for years. These symptoms are likely caused by an inflammatory process affecting the brachial plexus nerves. Branches derived from the upper and middle brachial plexus are most often involved. Additionally, phrenic nerve involvement is seen in some cases, leading to significant respiratory dysfunction presenting in a restrictive pattern. The sensory and motor symptoms do not follow a uniform anatomical course. The presentation varies from single nerve involvement to a patchy distribution.

PTS may be idiopathic or have a genetic predisposition, making the exact cause of the condition difficult to identify. Cases of PTS have been documented following a variety of inciting factors, including viral illness, vaccination, trauma, stress, or surgery, with a greater incidence noted among males. Due to its classic presentation, PTS is typically diagnosed clinically; however, electrodiagnostic studies, such as electromyography and nerve conduction studies, are used to confirm its diagnosis. While these studies may be normal initially, evidence of demyelination and axonal injury can be seen as symptoms progress and remain untreated. Therefore, it is important to treat patients as soon as PTS is identified to prevent nerve injury progression. Treatment modalities include corticosteroids, non-steroidal anti-inflammatory drugs, and opioids.

Though the symptoms and diagnosis of PTS have been well-described in the literature, the physical and temporal distribution of symptoms, along with its unclear etiology, result in this condition being frequently misdiagnosed. Notably, cases of postoperative PTS have been incorrectly attributed to perioperative nerve injuries due to positional traction or regional anesthesia. However, following perioperative neurologic injury, symptoms arise immediately, and pain is not typically followed by the prolonged muscle weakness seen in PTS. As such, understanding the distinction between symptoms of PTS and perioperative neurologic injury is imperative for those providing perioperative care, especially from a medicolegal perspective. Therefore, the purpose of this review is to describe PTS while highlighting the importance of its proper diagnosis, particularly in the postoperative setting.

## Introduction and background

Parsonage-Turner syndrome (PTS) is an uncommon disorder characterized by abrupt-onset unilateral shoulder girdle pain followed by sensory disturbances and upper limb weakness [1]. This constellation of symptoms was first documented in 1943 [2] and formally named by Parsonage and Turner in 1948 [1]. PTS has since been referred to as acute brachial plexopathy, brachial plexus neuritis, neuralgic amyotrophy, and idiopathic amyotrophic neuralgia, with patient presentation remaining the same [3]. Patients with PTS experience sudden, severe unilateral pain affecting the shoulder girdle and sometimes the arm, lasting from a few hours to up to four weeks. Two to four weeks after the onset of pain, patients may begin to experience upper limb weakness. Notably, this weakness can begin as arm pain subsides and may persist for years in some patients [4,5].

Early studies reported the incidence of PTS to be 1-3/100,000 cases [6], but recently, studies have shown an incidence closer to 1/1,000 cases [4,7]. While the exact etiology of PTS is unknown, it has been reported following several inciting factors, including viral illness, vaccination [8], trauma, stress, surgery, and a hereditary disposition to PTS, with a greater incidence in males than in females [3,5,9,10]. The wide spectrum of possible causes and clinical symptoms, coupled with the unclear pathophysiology of this syndrome, leads patients to seek care from a variety of practitioners, leading to delayed and sometimes incorrect diagnoses.

The array of symptoms seen in PTS is attributed to the varied involvement of brachial plexus nerves. While the upper and middle trunks are most commonly affected, PTS may also affect distal brachial plexus nerves. Phrenic nerve involvement can also be seen in up to 14% of cases [3,11]. The implications of general anesthesia on phrenic nerve blockade often lead to phrenic nerve involvement in PTS being misattributed to an anesthetic cause [5]. Further, regional anesthesia and positioning nerve injury have been incorrectly cited as the cause of postoperative PTS [3]. The ambiguous etiology of PTS and possible subsequent misdiagnosis of the condition as a result of a perioperative or anesthetic error is of particular importance to perioperative care providers [9]. This review aims to further describe PTS to anesthesiologists and other perioperative providers, given the medicolegal implications of postoperative neurological deficits. To synthesize this review, PubMed searches were performed, using the keywords “Parsonage-Turner syndrome,” “brachial plexopathy,” “brachial plexus neuritis,” “neuralgic amyotrophy,” and “idiopathic amyotrophic neuralgia,” with emphasis placed on publications released between the years 2000 and 2025. Publications such as narrative reviews, meta-analyses, randomized controlled trials, and case reports were included. Earlier publications are cited to provide relevant background information.

## Review

Epidemiology

While PTS is a well-documented condition, determining its exact cause, whether hereditary or idiopathic, remains a challenge [12]. Hereditary PTS is an autosomal dominant condition caused by a mutation in the SEPT9 gene on chromosome 17q25 [13]. Idiopathic PTS, however, may be caused by a wide variety of precipitating events or have no antecedent cause.

Etiology

A study of 246 cases reported that infection (45.5%), exercise (17.4%), and surgery (13.9%) were the most common factors preceding the onset of PTS symptoms, with labor/pregnancy, vaccination, psychological stress, or trauma seen less frequently [4]. It has also been proposed that PTS symptoms may be caused by activation of a dormant virus due to surgical stress or by an active viral trigger, such as group A streptococcus [10,14]. Additionally, autoimmune causes have been suggested, as positive IgM and IgG ganglioside antibodies have been found in patients experiencing postoperative PTS [10,15]. Further, cases of PTS have been seen in patients undergoing hematopoietic stem cell transplant, corroborating hypotheses favoring the immune-mediated onset of the syndrome [16]. The diverse causes of PTS, therefore, suggest that the pathophysiology of the condition is mediated by environmental, genetic, or immunological factors, an important fact for perioperative providers to be aware of [17].

Diagnosis

Regardless of the etiology, patients with PTS present in a similar manner, making clinical recognition and diagnosis of utmost importance. As most patients present with acute unilateral pain, diagnoses made in the initial phase of the clinical course commonly include radiculopathy or shoulder joint injury, leading to referrals to primary care physicians, neurologists, physiatrists, and orthopedic surgeons. The clinical picture is further complicated by neuropathic pain and motor weakness following the initial pain attack, which occurs in patchy distributions and differs between patients [18]. This distribution of symptoms, characterized by sensory and motor symptoms that do not necessarily affect the same nerves, is the hallmark of PTS and is key to proper diagnosis [18]. Cases of postoperative PTS have been incorrectly attributed to perioperative nerve injuries due to positional traction or regional anesthesia. However, following perioperative neurologic direct nerve injury, symptoms arise immediately, and pain is not typically followed by the prolonged muscle weakness seen in PTS.

Although PTS is mainly diagnosed clinically, electrodiagnostic studies help confirm the correct diagnosis [19]. It is not uncommon for patients to initially have normal nerve conduction studies. Treatment is crucial to prevent axonal loss and denervation. Nerve damage in PTS can have a mixed picture of demyelinating (neuropraxia) and/or axonal (axonotmesis) injury [4,19,20]. Although the inflammation causing focal nerve damage throughout the brachial plexus can be observed histologically [21,22], the clinical diagnosis of PTS is typically confirmed through electromyography (EMG) and nerve conduction studies. EMG has shown PTS to be an axonal process characterized by sharp positive waves and fibrillation potentials in nerves innervating the affected muscles [11].

Initial studies using EMG help localize affected nerves within the plexus and peripherally, as well as for monitoring improvement in nerve function [4]. Imaging may be indicated when the clinical picture is inconclusive or when symptoms persist despite treatment. Notably, hourglass-like constrictions are commonly seen in cases of PTS, appearing on ultrasound and magnetic resonance neurography in up to 88% of patients. Histologically, these constrictions reveal local or diffuse loss of myelin [23,24]. MRI of the brachial plexus may reveal nonspecific inflammation, although imaging of the affected muscles has demonstrated denervation [25]. MRI for cervical spine imaging must be used sparingly, however, as incidental, subclinical C5-C6 pathologies are common and may confound the clinician’s diagnostic ability [4].

Management

The management of PTS is dependent on the time of diagnosis and the location of the anatomical sites affected, and involves the use of both pharmacological and non-pharmacological methods. Historical data have shown shorter symptom durations in patients treated acutely with short courses of oral steroids, potentially decreasing inflammation in the affected nerves; however, evidence of definitive treatment is lacking [26]. A study by van Alfen and van Engelen suggested oral prednisone of 1 mg/kg for one week, followed by a taper in the second week. In another study, patients were treated with 60 mg of prednisolone for one week, followed by a 10 mg daily taper in the second week [26]. During the acute phase, characterized by predominantly musculoskeletal pain, conservative management (warm packs, avoiding movement) may provide relief. Patients have been shown to respond well to non-steroidal anti-inflammatory drugs and opioids when used either alone or in combination (i.e., slow-release diclofenac with morphine) [4]. Additionally, some studies have found positive results after using intravenous immunoglobulins [20]. Following the initial pain episode, a subacute phase develops, and patients typically develop neuropathic pain, therefore requiring multimodal pain management including neuropathic agents, especially in cases of chronic pain [27]. Patients may remain on their initial regimen and add first-line agents used to treat neuropathic pain, such as anticonvulsants (e.g., gabapentin, pregabalin), tricyclic antidepressants (e.g., amitriptyline, nortriptyline), or selective serotonin-norepinephrine reuptake inhibitors (e.g., duloxetine) [26,27]. If a chronic phase develops, the patient may require a referral for chronic pain management as well as surgical consultation [28].

In addition to pharmacological treatments, physical therapy may be used to treat PTS, including strength training, osteopathic or chiropractic manipulations, and exercises focused on stretching and increasing range of motion [26,29]. The ability of adaptive neuroplasticity to guide recovery through targeted rehabilitation efforts has also been investigated, with the NA-CONTROL trial being the first to study this [30]. The trial aims to examine the utility of relearning motor control and targeting cerebral mechanisms in improving impaired motor planning in the upper extremity [30].

Other modalities, such as acupuncture, transcutaneous electrical nerve stimulation, and epidural steroid injections, may also provide relief, though evidence is limited regarding their efficacy [26,31,32]. Other supportive measures, such as the use of braces for specific nerve injuries, may be warranted, depending on the severity of symptoms [28]. In refractory cases, surgical procedures may be considered, including neurolysis, nerve grafts, and nerve transfers, though these may lead to worse symptoms if performed beyond six months after initial presentation [26]. Notably, when the phrenic nerve is affected, patients should be screened for sleep disturbances and may require noninvasive ventilation, as this disorder behaves like a restrictive thoracic disease [28]. If patients remain symptomatic and no significant recovery of the phrenic nerve is evident within two to three years, surgical plication of the diaphragm may be considered [28].

Full recovery of motor and sensory symptoms is common; however, the time to recovery varies. In a study of 99 patients, 36% reported full symptomatic recovery within one year of initial pain onset, with 89% achieving full recovery within three years [33]. However, the small sample size of this study must be taken into consideration before extrapolating its findings. In another study of 246 patients, 78% showed recovery within the first year, and 24% achieved full recovery after one to three years [4]. However, a subset of patients may experience symptoms for a prolonged period, with one case describing symptoms that persist for over a decade and lead to subluxation of the glenohumeral joint [14]. The variation in the conclusions of these studies further emphasizes the diverse natural history of this syndrome, with each patient demonstrating a unique symptom course. Additionally, the absence of a large-scale randomized controlled trial analyzing different treatment modalities for PTS poses a challenge when adequately determining suitable symptom management.

Discussion

Perioperative neurologic injury may be attributed to positioning, patient factors, and surgical complications, with brachial plexopathy easily missed or misdiagnosed and attributed to anesthesia-related interventions or surgery-related complications. This is especially common when clinicians are unfamiliar with the diagnosis, making the distinction between brachial plexus injury and PTS of utmost importance [3].

Additionally, neurologic injury caused by peripheral nerve blocks has been incorrectly diagnosed in cases of PTS. However, it is extremely rare, with the incidence of injury reported to be two to four per 10,000 cases [34]. Neal et al. reported a case of neuralgic amyotrophy involving the phrenic nerve following right interscalene nerve blockade preoperatively for right shoulder arthroscopic rotator cuff repair. In this case, the injury was incorrectly attributed to the nerve block. The incorrect diagnosis is highlighted by several important factors later discovered, including prior patient history of idiopathic bilateral ulnar neuropathy, family history in his twin brother of multiple episodes of brachial plexopathy, and onset of brachial plexopathy in the contralateral upper extremity after undergoing contralateral shoulder arthroscopic repair years later without administration of a nerve block [3].

While some surgeons may be familiar with neuralgic amyotrophy, other perioperative providers may be unfamiliar with this syndrome and other postsurgical inflammatory neuropathies. Postsurgical PTS can be easily diagnosed by experienced providers when occurring at an anatomically distant site from where the surgery took place. However, diagnosis may become nebulous when the surgical site is on the ipsilateral extremity or cervical spine. If a cervical procedure was performed and brachial plexopathy/PTS is suspected, a surgical complication, such as C5 palsy, must be ruled out. As mentioned previously, a key feature that suggests PTS is a delay of several days in the onset of symptoms, while positioning or traction injuries are evident shortly after a procedure is completed. In PTS, pain is also typically disproportionate to the expected postoperative course. The clinical course is also very specific in that delayed severe pain is followed by a prolonged neurologic deficit weeks later. One case describes PTS in a patient who underwent lumbar spine surgery, with symptoms beginning in her right arm immediately after surgery and symptoms in her left arm beginning after a delayed period. The delayed nature of the pain suggests the presence of a source other than solely positioning, with further imaging by MRI and EMG revealing a diagnosis of PTS, allowing for proper and timely treatment [35]. Further large-scale studies comparing symptom onset, duration, and management are necessary to aid in the perioperative provider’s understanding of this syndrome.

## Conclusions

The constellation of symptoms seen in PTS can occur spontaneously or post-surgically, and effective management relies on early treatment, emphasizing the need for prompt recognition and diagnosis. As this rare syndrome is easily confused with other commonly seen surgical and anesthetic complications, anesthesiologists and other perioperative care providers must be familiar with its presentation. Additionally, though clinical diagnosis of PTS is possible, a proper diagnostic assessment should be performed to distinguish between PTS and other perioperative complications.
